# Reply to Comment by Zhang on “Exploring the Influence of Smallholders' Perceptions Regarding Water Availability on Crop Choice and Water Allocation Through Socio‐hydrological Modeling”

**DOI:** 10.1029/2018WR024328

**Published:** 2019-03-26

**Authors:** L. Kuil, T. Evans, P. F. McCord, J. L. Salinas, G. Blöschl

**Affiliations:** ^1^ Centre for Water Resource Systems Vienna University of Technology Vienna Austria; ^2^ School of Geography and Development University of Arizona Tucson AZ USA; ^3^ Center for Systems Integration and Sustainability Michigan State University East Lansing MI USA; ^4^ Institute of Hydraulic Engineering and Water Resources Management Vienna University of Technology Vienna Austria

## Abstract

Zhang (2019, https://doi.org/10.1002/wrcr.v54.4) criticizes several of the assumptions and parameter choices of the model of Kuil et al. (2018, https://doi.org/10.1002/2017WR021420) and claims that, due to an inconsistency in the irrigation equation, the key findings should be interpreted with much caution. We address each of the comments and show that the conclusions of Kuil et al. (2018, https://doi.org/10.1002/2017WR021420) remain fully valid.

## Key Points

- We show that the assumptions and parameter choices can be justified in the light of the purpose of the model and the existing literature
- We demonstrate that the conclusions presented in Kuil et al. (2018) remain fully valid
- We maintain that the results are fully replicable if a similar amount of irrigation is applied

## 1 Introduction

The original paper by Kuil et al. (2018) explored the role of farmers' perceptions of water availability on crop choice and water allocation. To this end, a stylized model was developed following a socio‐hydrological approach (Sivapalan et al., 2012; Sivapalan & Blöschl, 2015). Central to the model framework are the interactions between farmers and their environment, focusing on the potential mechanisms behind observed behavior. Zhang (2019) raises several concerns regarding the model which are addressed below.

## 2 Socio‐hydrological Model

### 2.1 Assumptions

As a first point, Zhang (2019) notes that deep percolation from the bottom soil layer is not included in the model even though deep percolation could contribute a significant proportion of the irrigation water losses.

It is correct that deep percolation is not included as a separate term in the water balance equation, represented in Kuil et al. (2018) as follows:

(1) $$\frac{dS_{1,2}}{dt}=P+I_{1,2}-T_{1,2}-E_{1,2}-Q_{1,2}$$

Generally, deep drainage forms a relatively small component of the annual water balance and is frequently less than the error in any estimate of actual evapotranspiration (Walker & Zhang, 2002). Therefore, unless the actual goal of the model is the modeling of deep percolation, inclusion of this process without substantial data for validation will make the model more complex without making it more informative. In view of the model's purpose to capture the main interactions between farmers and their environment (and not to represent the hydrological processes in all its detail) a single layer bucket model, where all excess water is captured by the runoff term, is deemed sufficient. By doing so, we believe that model complexity is in line with model purpose as suggested by Zhang et al. (2002) and Troy et al. (2015), among others. In addition, the case study of Kuil et al. (2018) is the Upper Ewaso N'giro basin which lies at the foot of Mount Kenya and consists of predominantly sloping terrain with clayey soils, conditions that are not conducive to deep percolation (Bethune et al., 2008). In case additional complexity was desired, deep percolation could of course be included, but, because of the small contribution to the water balance, the fundamental behavior of the model and therefore the conclusions of the paper are not expected to change.

The second point of Zhang (2019) is that transpiration and evaporation were simulated separately. He notes that these two processes are very difficult to separate in reality and considers the assumptions of negligible evaporation during the growing season and negligible transpiration during the off‐season inappropriate.

The aim of the study was not to model the *partitioning* of evaporation and transpiration in crop lands but the overall loss of water to the atmosphere. Consequently, considering the stylized nature of the model, simplifying choices have been made and only the dominant processes, that is, transpiration during the growing season and evaporation during the dry season (FAO, 1998), have been included in the equations. During the growing season, bare soil evaporation usually is much lower than transpiration, and outside the crop season the opposite is the case (Ritchie, 1972; Villalobos & Fereres, 1990) suggesting that the assumptions made are appropriate. Again, more complexity could be introduced, but the fundamental behavior of the model is not expected to change.

Third, Zhang (2019) points out that equation (1b) of Kuil et al. (2018) should be replaced by

(2) $$I_{1}=D\;\frac{1}{C_{1}}\left(I-C_{2}I_{2}\right)$$

where *I* is the total irrigation water, *I*_1_ and *I*_2_ are the irrigation water for crops 1 and 2, respectively, and *C*_1_ and *C*_2_ are the fractions of the crops. Equation (2) is indeed more consistent and should be preferred. When using equation (2) instead of equation (1b) of Kuil et al. (2018), crop 1 will receive more irrigation water. While this change affects soil moisture levels, memory levels, and yield (especially of crop 1) as Zhang also points out in section 2.3 (Zhang, 2019), it does not impact the model's fundamental behavior. To support this statement, we reran the model with equation (2). The results are presented in section 2.3.

### 2.2 Parameters

Regarding equation (3) (equation (1e) of Kuil et al., 2018)

(3) $$Q_{1}=\frac{P+I_{1}}{1+e^{\varepsilon_{H}\left(\xi_{H}-\frac{S_{1}}{\varphi_{H}}\right)}}$$

where *Q*_1_ is runoff from the area allotted to crop 1, *P* is precipitation, *I*_1_ is the irrigation water allocated to crop 1, *S*_1_ is the soil moisture per unit area for crop 1, and *φ*_*H*_ is field capacity. Zhang (2019) points out that the values of the parameters *ε*_*H*_ and *ξ*_*H*_in the text, section 5.2 of Kuil et al. (2018) must be erroneously provided as they do not lead to runoff regardless of how large antecedent soil moisture is.

**Figure 1:**
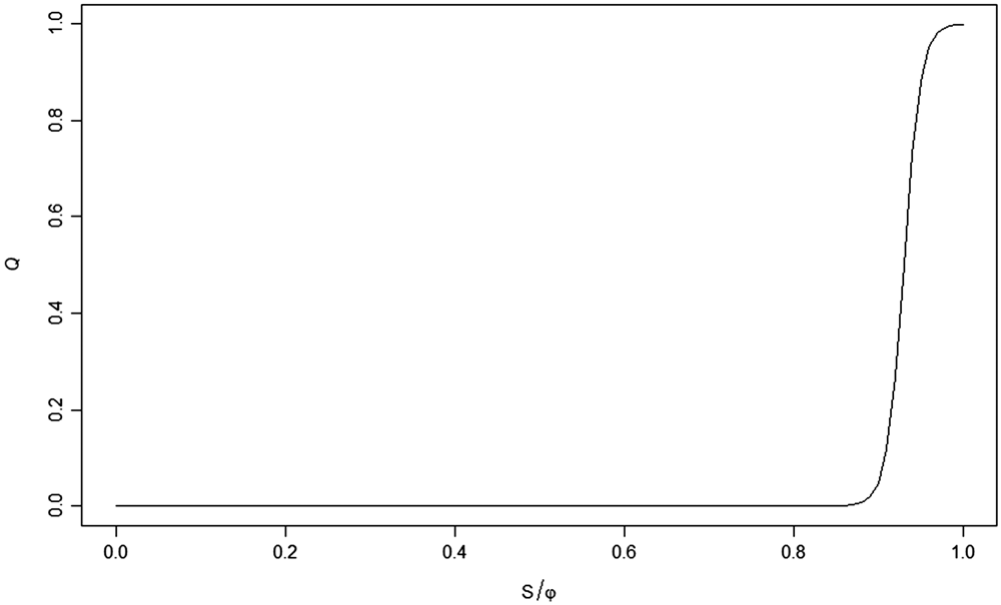
Relationship between runoff (Q) and S/φ_H_ for the parameter value ε_H_ = 100 and ξ_H_ = 0.93.

The values in the text have been inadvertently swapped, and the correct values are the ones stated in Table 1, that is, *ε*_*H*_ = 100 and *ξ*_*H*_=0.93, of Kuil et al. (2018). Model results are consistent with the parameters of Table 1, which do represent a hydrologically realistic situation. As noted by Kuil et al. (2018), the parameters were set to reflect an irrigation situation, that is, a situation optimal for water to reach the soil. Therefore, it is assumed that the soil storage first fills up to a large extent before runoff occurs (see Figure 1).

We acknowledge that the reality may be more complex, as infiltration may depend on many factors, including soil texture, clay mineralogy, crop and land management practices, soil water content, and rainfall intensity (Blackburn, 1975). However, in light of the overall purposes of the model, the current representation was deemed appropriate. A more detailed representation of the processes is not expected to significantly alter the main feedbacks of the model framework nor the conclusions of the paper.

In the next comment, Zhang observes that the initial values of Memory values *M*_1_ and *M*_2_ are different when comparing Figure 6b to Figure 6a. While this modification does not influence the overall calibration run—solely the initial readjustment of the model—it is indeed slightly more accurate when the initial values for both *M*_1_ and *M*_2_ are kept at 1. When rerunning the model with equation (2), memory values have therefore been kept at 1 for the calibration run (see Figure 3, section 2.3).

Subsequently, Zhang observes that the calibrated model simulated a slower increasing trend of the fraction of crop with increasing water availability than the uncalibrated model (comparison of Figures 6b and 6a, second row). This can indeed be observed. Zhang suggests that this is due to modification of crop adaptation parameter *α*_C_, which determines the speed at which farmers change the proportion of crops in response to their perception of water deficit. This is not correct.

As has been stated in Kuil et al., 2018, the difference between Figures 6b and 6a, are the management parameters. These management parameters influence how the irrigation water is allocated and when a farmer chooses to increase or decrease his/her crop fraction *C*_2_. To understand this better, we might look, for example, at the equation governing crop decision making (equation (4))

(4) $$\frac{{dC}_{2}}{dt}=\left(1-D\right)\left(\alpha_{C}\left(\left(\beta_{C}M_{1}+\gamma_{C}\right)-M_{2}\right)C_{2}\left(1-C_{2}\right)\right)$$

Here *C*_2_ represents the crop fraction of *C*_2_. *D* is a dummy variable equal to 1 during the growing season and equal to 0 outside of the growing season. *α*_*C*_ [*T* ^−1^] determines the rate at which a farmer changes crop type as a consequence of changes in memory. *β*_*C*_ [−] and *γ*_*C*_ [−] represent management strategy parameters.

The equation is a simple logistic equation, where the crop fraction of *C*_2_ will decrease if (1 − *D*)(*α*_*C*_((*β*_*C*_*M*_1_ + *γ*_*C*_) − *M*_2_)*C*_2_(1 − *C*_2_)) is negative. We focus on (*β*_*C*_*M*_1_ + *γ*_*C*_) − *M*_2_. With the parameter values corresponding to Figure 6a, that is, ((1 * *M*_1_ + 0) − *M*_2_), this equation reduces to *M*_1_ = *M*_2_ for d*C*/d*t* = 0. However, when using the parameter values corresponding to Figure 6b, that is, ((0.6 * *M*_1_ + 0.2) − *M*_2_), this results in $M_{1}=\frac{M_{2}-0.2}{0.6}$. This means that, when there is limited water available and *M*_2_ > 0.5, the farmer acts as if the deficit of crop 2 were even higher. For the same amount of irrigation water available, the farmer will therefore reduce his/her *C*_2_ crop area, such that a higher irrigation water depth is reached per *C*_2_ area and the balance between *M*_1_ and *M*_2_, as the farmer perceives it, is restored. This is what is observed in Figure 6b. This decision is made irrespective of the value *α*_*C*_, as this does not determine when ((*β*_*C*_*M*_1_ + *γ*_*C*_) − *M*_2_) equals 0.

### 2.3 Model Results When Using the More Consistent Irrigation Equation

This section presents the results we obtained after we reran the model with equation (2) and recalibrated it. Hereby we set the management parameter *β*_*C*_ to 0.6 (was 0.4). The initial values of *M*_1_ and *M*_2_ are now kept at 1.

**Figure 2:**
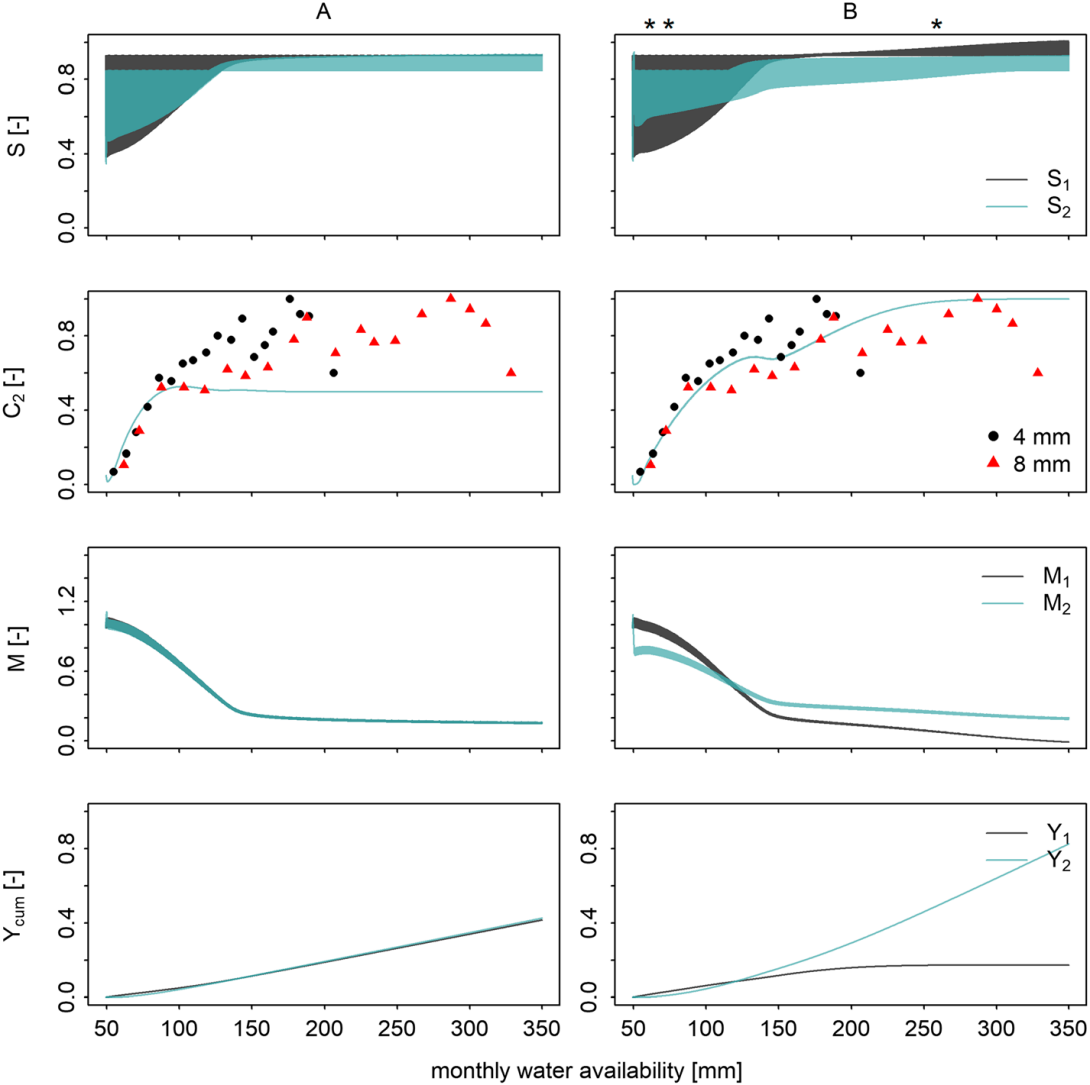
Simulation results for an (a) uncalibrated model and (b) a calibrated model. (from top to bottom) Soil moisture S_1,2_ (scaled by maximum water content), drought‐intolerant crop fraction C_2_, drought memory M_1,2_, and cumulative yield Y_cum_ (scaled by the maximum of cumulative yield. The simulations (each lasting 1,000 years) are mapped onto a water availability range from 50 to 350 mm/month. While drought memory of both crops remain close to each other in case simulation (third row), drought memory of crop 2 is kept low at the cost of drought memory of crop 1 in case of simulation (b), indicating a clear water allocation preference for crop 2 when water availability is scarce. The width of S in the first row represents seasonal variability.

Figure 2 shows the results of the calibration. As compared to Figure 6 of Kuil et al. (2018), soil moisture levels of crop 1 are higher as there is more irrigation water to crop 1, memory is lower, and the cumulative yield of crop 1 is higher. However, the overall behavior remains similar. As in Figure 6, drought memory of crop 2 is kept low at the cost of drought memory of crop 1 in case of the calibrated simulation (Figure 6b, third row), indicating a clear water allocation preference for crop 2 when water availability is scarce.

**Figure 3:**
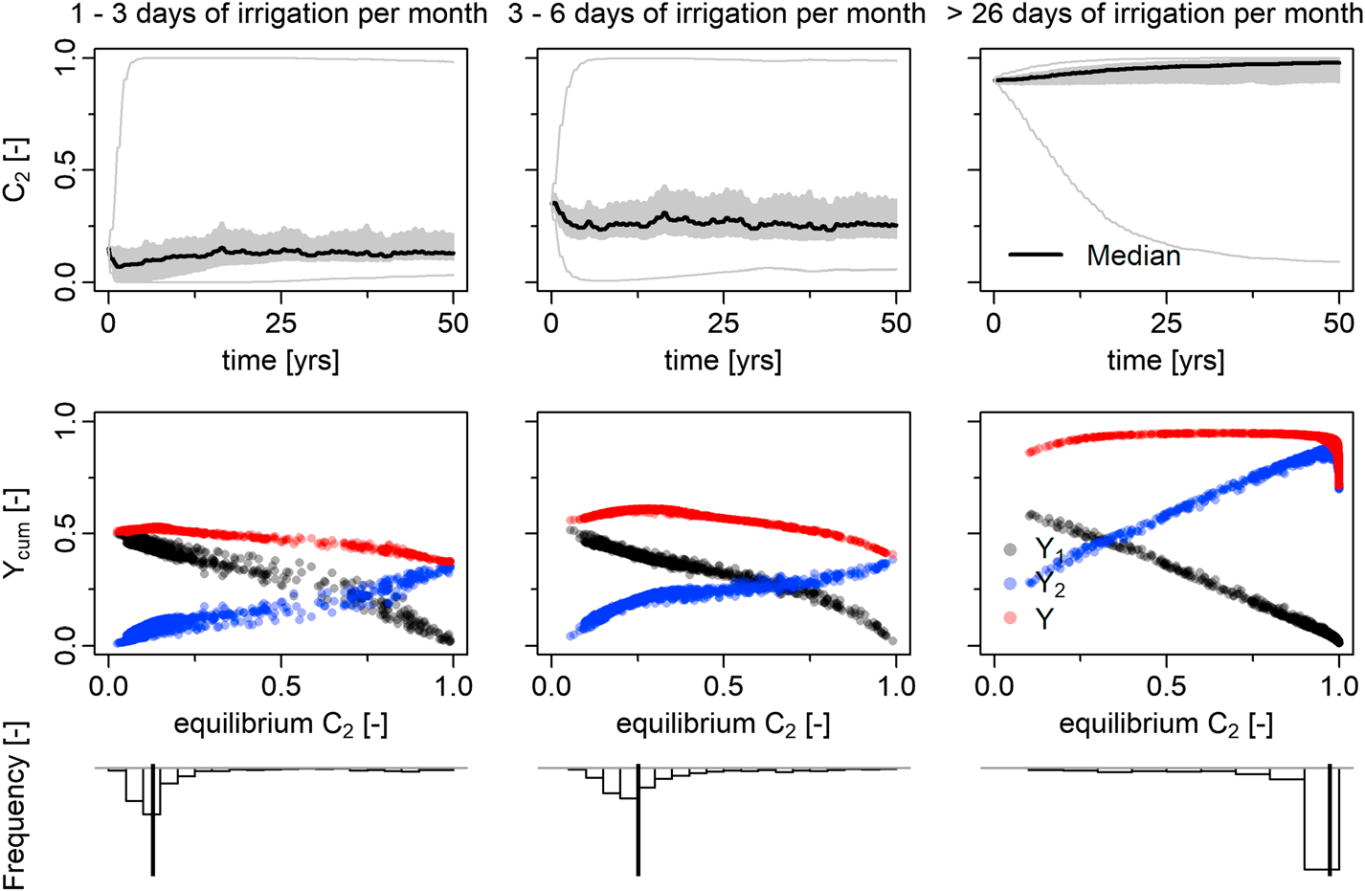
Outcome of the sensitivity analysis varying management parameter β_C_, memory loss rate β_M2_, and memory encoding parameter α_M2_. Each column summarizes the outcome of 1,000 simulations for a different level of monthly irrigation water availability, that is,, 1–3 irrigation days, 3–6 irrigation days, and >26 irrigation days per month. Precipitation is stochastically generated but kept the same for all simulations. (first row) Drought‐intolerant crop fraction C_2_ versus time. The median (bold black line), 25th and 75th quantiles (gray band), and minimum and maximum values are presented. (second row) Cumulative yield for Y_1_, Y_2_, and Y (=Y_1_ + Y_2_) versus the equilibrium crop pattern for each simulation. (third row) Histogram showing the marginal distribution of equilibrium C_2_.

As compared to Figure 7 of Kuil et al. (2018), the sociosensitivity analysis of Figure 3 shows somewhat increased cumulative yields (second row) after 50 years of farming for crop 1 as a result of increased irrigation water to crop 1, but, again, the basic model behavior remains unchanged. In both cases, the results show that there is an optimum range of crop fractions *C*_2_ with highest yields. When irrigation water availability is low (left and middle columns of Figure 3), this optimum occurs at low fractions of drought intolerant crop *C*_2_. When irrigation water is abundant, crop 2 is preferred and crop fraction *C*_2_ goes to 1. Moreover, the majority of the farmers, represented by their own unique combination of a memory encoding rate, memory loss rate, and a management strategy, are able to find this optimum (third row of Figure 3).

**Figure 4:**
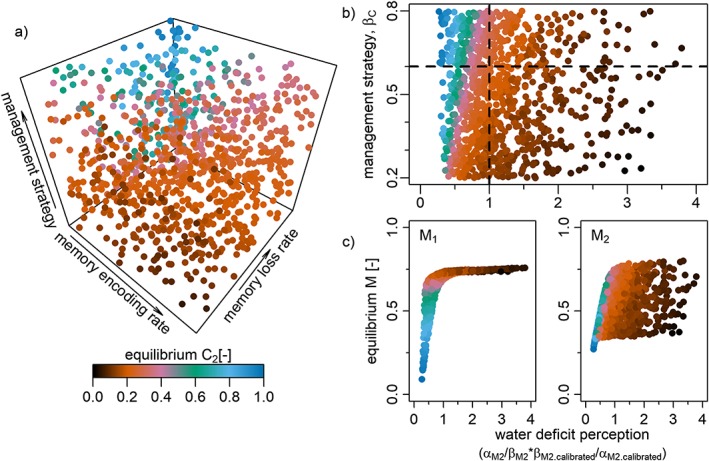
The effect of perceived water deficit level and management strategy on equilibrium crop C_2_ (drought‐intolerant crop fraction). Results are shown for 3–6 days of irrigation water availability per month (corresponding to the middle column in Figure 3). (a) The effect of management strategy β_C_, memory loss rate β_M2_, and memory encoding parameter α_M2_ on equilibrium C_2_. (b) The effect of management strategy and perceived water deficit on equilibrium C_2_ (higher values imply that the water deficit of crop 2 is more strongly perceived). The intersection of the horizontal and vertical lines in plot (b) marks equilibrium C_2_ corresponding to the calibrated parameter combination. (c) Equilibrium memory M_1,2_ associated with equilibrium C_2_ levels, plotted against perceived water deficit.

Similar to Figure 8a of Kuil et al. (2018), Figure 4a gives the largest area of drought‐intolerant crop (equilibrium *C*_2_ approaches one, blue colors) when management parameter (*β*_*C*_) is high, memory encoding (*α*_*M*2_) for crop 2 is low and memory loss (*β*_*M*2_) is high. Similar to Figure 8b, color bands emerge when the perception of water deficit is plotted against the management strategy, underlining the conclusion that even though each farmer perceives the world a little differently (effectively underestimates or overestimates water availability), similar (near‐optimal crop patterns) can emerge, as a result of the interplay of a farmer's perception and the management strategy adopted. Figure 4c gives slightly lower memory levels than Figure 8c of Kuil et al. (2018) and the memory levels are less spread out, which is a consequence of the increase of irrigation water. More importantly, the overall pattern of the relationship between *M*_1_ and *M*_2_ in relation to the perceived water deficit remains unchanged. For blue colors to emerge (*C*_2_ approaches 1), water deficit is perceived to be low, while for brown colors to emerge (*C*_2_ approaches 0) water deficit is perceived to be high and crop 2 is preferred to crop 1 (*M*_2_ *< M*_1_ *)*.

**Figure 5:**
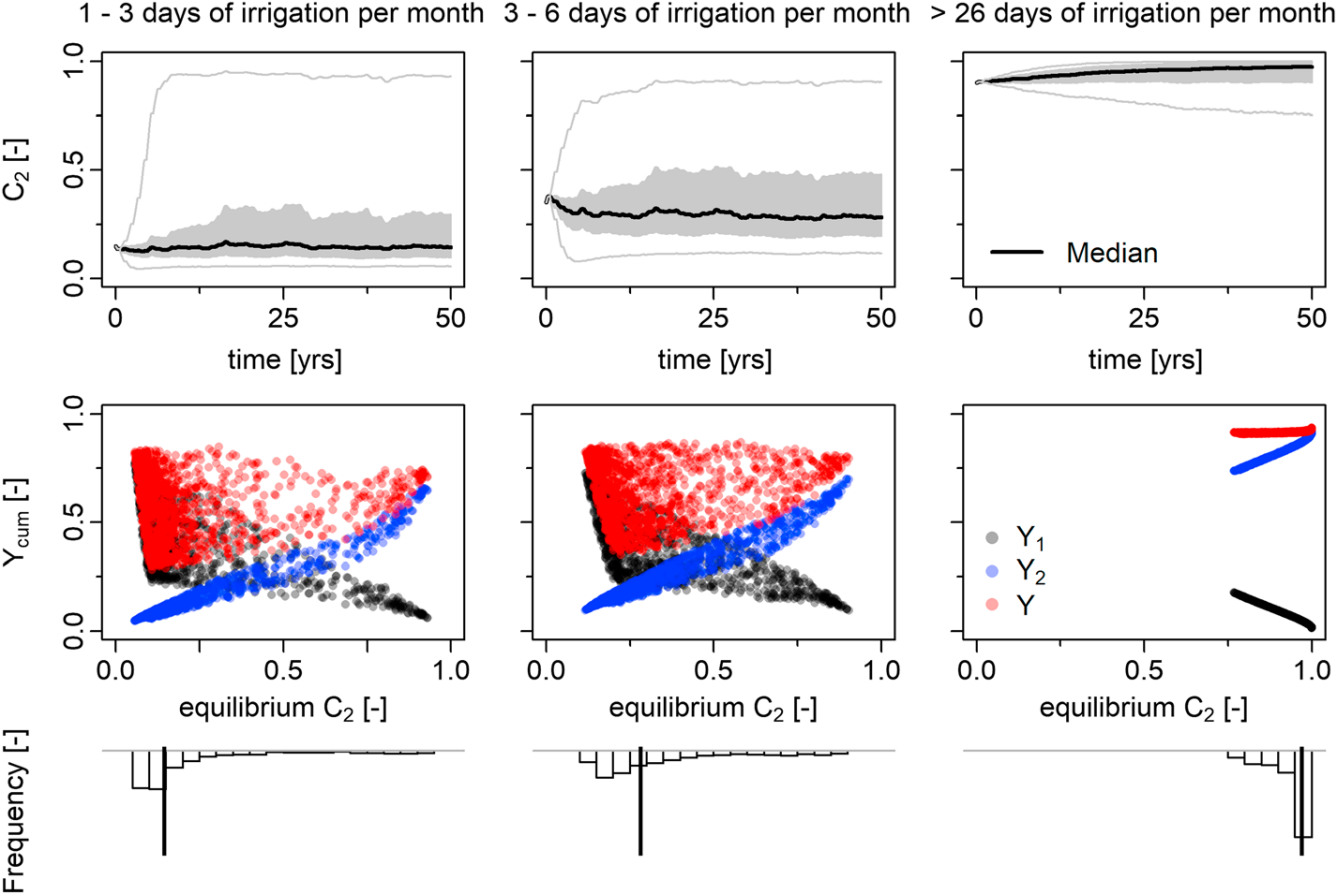
Outcome of the sensitivity analysis to maximum transpiration rate of crop 2, α_H2_, the maximum transpiration of crop 1, α_H1_, and the maximum soil moisture content, φ_H_. Each column summarizes the outcome of 1,000 simulations for a different level of monthly irrigation water availability. Precipitation is stochastically generated but kept the same during all simulations. (first row) Drought‐intolerant crop fraction C_2_ versus time. The median (bold black line), 25th and 75th quantiles (gray band), and minimum and maximum values are presented. (second row) Cumulative yield for Y_1_, Y_2_, and Y (= Y_1_ + Y_2_) versus the equilibrium crop pattern for each simulation. (third row) Histogram showing the marginal distribution of equilibrium C_2_.

Similarly to Figure 9 of Kuil et al. (2018), the hydrosensitivity analysis in Figure 5 shows more variation in yield than the sociosensitivity analysis (Figure 7 of Kuil et al., 2018, and Figure 3) since two of the three parameters directly influence the amount of water that is needed to produce one unit of yield, and most of this yield is due to variation in *Y*_1_. Due to the increased irrigation water availability to crop 1, the yields for crop 1 are somewhat higher than in Kuil et al. (2018).

**Figure 6:**
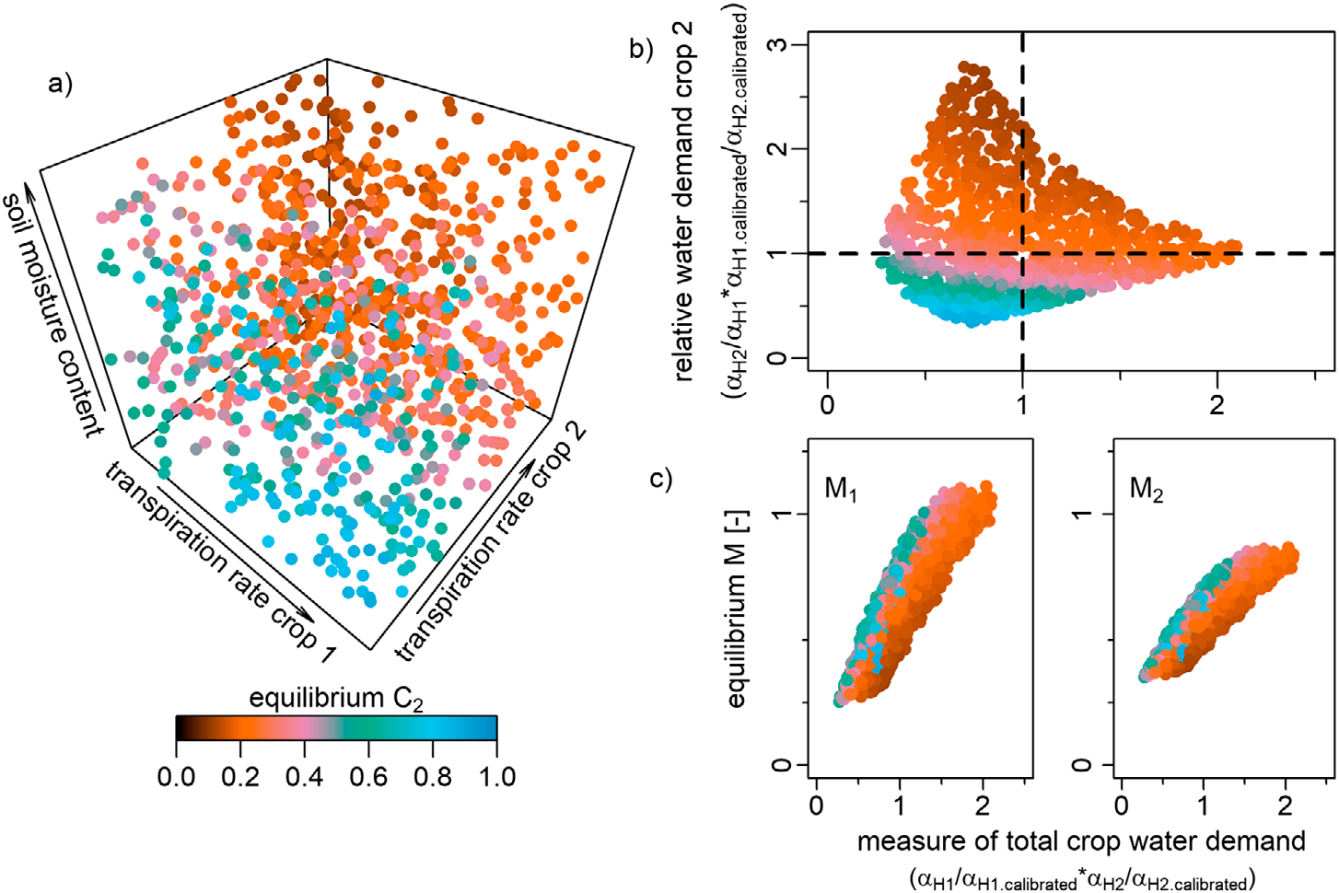
The effect of hydrological variability on equilibrium C_2_ (drought‐intolerant crop fraction). Results are shown for 3–6 days of irrigation water availability per month (corresponding to middle column, Figure 5). (a) The effect of varying maximum transpiration rate of crop 2, α_H2_, maximum transpiration rate of crop 1, α_H1_, and maximum soil moisture content, φ_H._, on equilibrium C_2_. (b) The effect of relative water demand and total crop water demand on equilibrium C_2_. The intersection of the horizontal and vertical lines in plot (b) marks equilibrium C_2_ corresponding to the calibrated parameter combination. (c) Equilibrium memory M_1,2_ associated with equilibrium C_2_ levels and plotted against the degree of total crop water demand.

Similar to Figure 10a of Kuil et al. (2018), Figure 6a shows that the area allocated to the drought‐intolerant crop (equilibrium *C*_2_ approaches one, blue colors) increases when the maximum transpiration rate of crop 2 (*α*_*H*2_) decreases or when the maximum transpiration rate of crop 1 (*α*_*H*1_) increases. The area is dominated by crop 2 (blue colors), when the maximum transpiration rate of crop 2 is less than the transpiration rate of crop 1 (relative water demand <1), and when total crop water demand is low (Figure 6b). The overall memory levels in Figure 6c are somewhat lower than those in Figure 10c of Kuil et al. (2018) due to the increased irrigation water to crop 1, but the overall patterns of a positive relationship between total crop water demand and increasing drought memory, and a divergence in drought memory (*M*_1_ > *M*_2_) of both crops as total water demand increases, remain the same.

**Figure 7:**
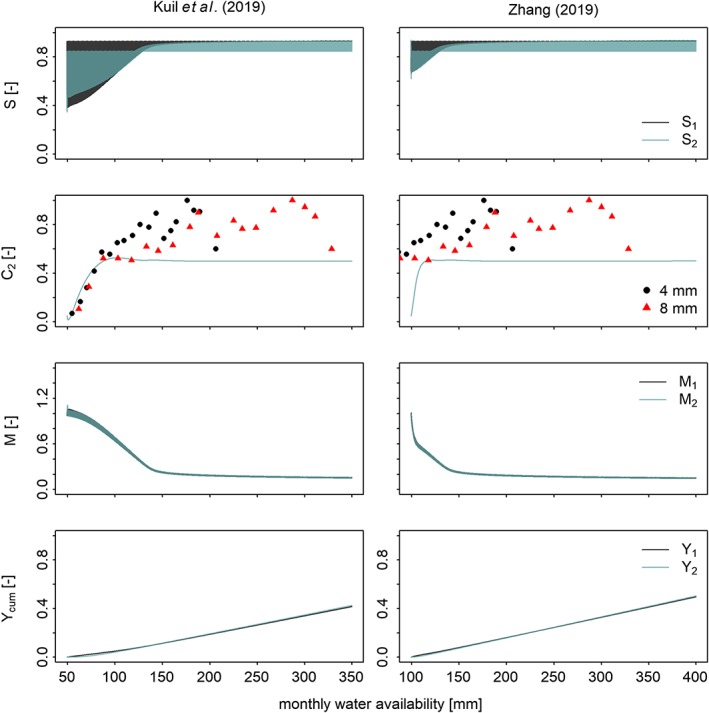
(left column) Simulation results for the uncalibrated model when subject to an irrigation flux that increases from 0 to 301 mm (see also Figure 2a of Kuil et al., 2019). (right) Simulation results for the uncalibrated model when subject to an irrigation flux that increases from 50 to 350 mm similarly to what has been coded by Zhang (2019).

With the figures above we have demonstrated that the model's fundamental behavior has not changed after adjusting the irrigation equation. Also, Zhang (2019) is able to generate comparable results, the exception being the shorter time period of the state variables *S*_1_, *S*_2_, *M*_1_, *M*_2_, and *C*_2_ to reach an equilibrium state. Zhang lists three possible reasons that could explain this difference, that is, (i) differences in numerical algorithm to solve the social‐hydrological model, (ii) differences in parameter values, or (iii) the presence of additional conditions in the modeling framework by Kuil et al. (2018). Based on the code Zhang provided on github (committed 9 December 2018), we like to offer an alternative explanation to the above possibilities. The amount of water that is available during the simulation is based on the amount of precipitation and irrigation that is available during a time step. In both the model of Kuil et al. (2018) and Zhang (2019) precipitation *P* is constant at 49 mm. Irrigation *I*, however, increases from 0 to 301 mm in Kuil et al. (2018), but from 50 to 350 mm in the code provided by Zhang (2019). As much more water is available during the beginning of the simulation in the model of Zhang (2019), it makes sense that the state variables converge much faster to equilibrium values. To demonstrate that the difference between the results can indeed be explained due to the difference in irrigation water, we ran our model while increasing the irrigation water from 50 to 350 as Zhang (2019) has done. The result is presented in Figure 7.

## 3 Conclusion

This paper has addressed each of the points raised by Zhang (2019). We have shown that the assumptions and parameters of Kuil et al. (2018) claimed by Zhang (2019) to be inappropriate or wrong are model choices that can be justified in light of the purpose of the model and the existing literature. Zhang (2019) suggested equation (1b) of Kuil et al. (2018) to be replaced by equation (2). We agree that this equation is more consistent, but it does not lead to a major change in model behavior, and the conclusions of Kuil et al. (2018) remain fully valid. The difference in results between Zhang (2019) and Kuil et al. (2018) can be explained by a difference in the irrigation flux, as Zhang (2019) assumed this increases from 50 to 350 mm during the simulation time, while this increases from 0 to 301 mm in the simulation by Kuil et al. (2018).

## References

[wrcr23866-bib-0001] Bethune, M. G. , Selle, B. , & Wang, Q. J. (2008). Understanding and predicting deep percolation under surface irrigation. Water Resources Research, 44, W12430 10.1029/2007WR006380

[wrcr23866-bib-0002] Blackburn, W. H. (1975). Factors influencing infiltration and sediment production of semiarid rangelands in Nevada. Water Resources Research, 11(6), 929–937. 10.1029/WR011i006p00929

[wrcr23866-bib-0004] FAO , (1998). Chapter 5 – Introduction to crop evapotranspiration. *Crop evapotranspiration ‐ Guidelines for computing crop water requirements ‐ FAO Irrigation and drainage paper* 56. Retrieved from http://www.fao.org/docrep/X0490E/x0490e0a.htm#single%20and%20dual%20crop%20coefficient%20approaches

[wrcr23866-bib-0005] Kuil, L. , Evans, T. , McCord, P. F. , Salinas, J. L. , & Blöschl, G. (2018). Exploring the influence of smallholders' perceptions regarding water availability on crop choice and water allocation through socio‐hydrological modeling. Water Resources Research, 54, 2580–2604. 10.1002/2017WR021420 PMC6559291 31217644

[wrcr23866-bib-1005] Kuil, L. , Evans, T. , McCord, P. F. , Salinas, J. L. , & Blöschl, G. (2019). Reply to Comment by Zhang on “Exploring the influence of smallholders'perceptions regarding water availability on crop choice and water allocation through socio‐hydrological modeling. Water Resources Research, 55 10.1029/2018WR024328 PMC6559291 31217644

[wrcr23866-bib-0006] Ritchie, J. T. (1972). Model for predicting evaporation from a row crop with incomplete cover. Water Resources Research, 8(5), 1204–1213. 10.1029/WR008i005p01204

[wrcr23866-bib-0007] Sivapalan, M. , & Blöschl, G. (2015). Time scale interactions and the coevolution of humans and water. Water Resources Research, 51, 6988–7022. 10.1002/2015WR017896

[wrcr23866-bib-0008] Sivapalan, M. , Savenije, H. H. G. , & Blöschl, G. (2012). Socio‐hydrology: A new science of people and water. Hydrological Processes, 26(8), 1270–1276. 10.1002/hyp.8426

[wrcr23866-bib-0010] Troy, T. J. , Pavao‐Zuckerman, M. , & Evans, T. P. (2015). Debates—Perspectives on socio‐hydrology: Socio‐hydrologic modeling: Tradeoffs, hypothesis testing, and validation. Water Resources Research, 51, 4806–4814. 10.1002/2015WR017046

[wrcr23866-bib-0011] Villalobos, F. J. , & Fereres, E. (1990). Evaporation measurements beneath corn, cotton, and sunflower canopies. Agronomy Journal, 82(6), 1153–1159. 10.2134/agronj1990.00021962008200060026x

[wrcr23866-bib-0012] Walker, G. R. , & Zhang, L. (2002). Plot scale models and their application to recharge studies. Collingwood, VIC, Australia: CSIRO Publishing 10.1071/9780643105423

[wrcr23866-bib-0013] Zhang, L. (2019). Comment on Exploring the influence of smallholders' perceptions regarding water availability on crop choice and water allocation through socio‐hydrological modeling by Kuil et al. Water Resources Research, 54, 2580–2604. 10.1002/wrcr.v54.4 PMC6559291 31217644

[wrcr23866-bib-0014] Zhang, L. , Walker, G. R. , & Dawes, W. R. (2002). Water balance modelling: Concepts and applications. ACIAR Monograph Series, 84, 31–47.

